# Research on Small Sample Dynamic Human Ear Recognition Based on Deep Learning

**DOI:** 10.3390/s22051718

**Published:** 2022-02-22

**Authors:** Yanmin Lei, Junru Qian, Dong Pan, Tingfa Xu

**Affiliations:** 1 Department of Electrical and Information Engineering, Changchun University, Changchun 130012, China; 0451lym@163.com (Y.L.); pandong791021@126.com (D.P.); 2 Jilin Province Key Laboratory of Measuring Instrument and Technology, Jilin Institute of Metrology, Changchun 130022, China; 3 School of Optics and Photonics, Beijing Institute of Technology, Beijing 100081, China; ciom_xtf1@bit.edu.cn

**Keywords:** deep learning, dynamic, ear recognition, small sample, multi-posture changes

## Abstract

Due to the problem of insufficient dynamic human ear data, the Changchun University dynamic human ear (CCU-DE) database, which is a small sample human ear database, was developed in this study. The database fully considers the various complex situations and posture changes of human ear images, such as translation angle, rotation angle, illumination change, occlusion and interference, etc., making the research of dynamic human ear recognition closer to complex real-life situations, and increasing the applicability of human ear dynamic recognition. In order to test the practicability and effectiveness of the developed CCU-DE small sample database, we designed a dynamic human ear recognition system block diagram based on a deep learning model, which was pre-trained by a migration learning method. Aiming at multi-posture changes under different contrasts, translation and rotation motions, and with or without occlusion, simulation studies were conducted using the CCU-DE small sample database and different deep learning models, such as YOLOv3, YOLOv4, YOLOv5, Faster R-CNN, and SSD. The experimental results showed that the CCU-DE database can be well used for dynamic ear recognition, and it can be tested by using different deep learning models with higher test accuracy.

## 1. Introduction

With the acceleration of the informatization process, the fields of information security, social security, national security, and financial transaction security have increasingly higher requirements for the accuracy of personal identity verification. However, the shortcomings of the existing traditional identity authentication methods, in terms of speed, accuracy, and security, are becoming more and more obvious, and it has gradually become more difficult to meet the needs of individuals and society. The rapid development of machine vision, big data and artificial intelligence provides favorable conditions for the development of biometric recognition technology. Therefore, the importance of biometric identification technology in ensuring safety technology is becoming more and more prominent. Existing biometric recognition mainly comprises human face recognition [1], voice recognition [2], fingerprint recognition [3], and iris recognition [4]. Each method has advantages and disadvantages, and has suitable fields of application. Human ears have gradually attracted the attention of relevant scientific researchers due to their characteristics of stability, non-invasiveness, expressionlessness, and significant individual differences [5]. In the current worldwide outbreak of COVID-19, ear recognition research is particularly important because people often wear masks.

The difference between human ear recognition and human ear detection is that the former can not only detect the human ear in the picture, but also determine whose ear it is; thus, human identity can be verified through human ear recognition.

At present, the methods of human ear recognition can be roughly divided into traditional methods, and methods based on deep learning. Since the recognition results of human ears are easily affected by changes in environment and posture, methods based on deep learning have more advantages. Galdamez et al. used convolutional neural network technology to study human ear recognition for the first time, and this research method was certainly groundbreaking [6]; Zhang Yi proposed a robust multi-scale Faster-R-CNN ear detection algorithm [7]; Priyadharshini et al. proposed a six-layer-deep convolutional neural network architecture for ear recognition [8]; Zhang et al. proposed a human ear recognition model based on the convolutional neural network [9]; Mursalin M. proposed higher localization 3D ear detection by deep learning [10]; and Shruti N. and Hemant K. M. have presented an approach towards using the ear as a biometric for identification based on deep learning, giving promising results [11]. At present, deep learning models for human ear recognition mainly include two-stage Faster-R-CNN and single-stage SSD (Single-Shot MultiBox Detector) and YOLO series. The YOLO series uses the idea of system regression to predict the category of the image. Compared with the two-stage target detection algorithm, the speed was faster [12].

Through the literature research, we can see that all the above methods can achieve static human ear recognition. However, there are two main problems in human ear recognition: (1) existing human ear detection and ear recognition are mostly static, but in practical applications, human ears are mostly in dynamic and unstructured environments; (2) existing human ear databases such as IITD [13], USTB [14], USTB-Helloear [7], AMI and EarVN1.0 [15], etc., are static.

In this paper, we mainly studied dynamic human ear recognition based on deep learning. The so-called dynamic human ear recognition was used to recognize the human ear in our recorded videos. Compared with human ear recognition in static pictures, in videos the human ear dynamically changes frame by frame, so it belongs to dynamic human ear recognition. We set up two hypotheses: (1) dynamic human ear recognition is affected by contrast, posture changes, illumination change, the size of translation angle or rotation angle, occlusion and interference; (2) different deep learning models such as the YOLO series (YOLOv3 [16], YOLOv4 [17] and YOLOv5 [18]), Faster R-CNN and SSD can be used to test the effectiveness of the dynamic human ear database.

The main contributions of this paper are as follows: (1) we developed a dynamic human ear database named CCU-DE; (2) we designed a dynamic human ear recognition system block diagram based on a deep learning model which was pre-trained by adopting the migration learning method; (3) we used various deep learning models for ear recognition to test the dynamic database CCU-DE.

The rest of the paper is arranged as follows. The block diagram of the dynamic human ear recognition system based on deep learning is outlined in Section 2. The development of the CCU-DE small sample database is introduced in Section 3. The experiment designs are presented in Section 4. The paper is summarized in Section 5.

## 2. Dynamic Human Ear Recognition System Block Diagram

**Figure 1:**
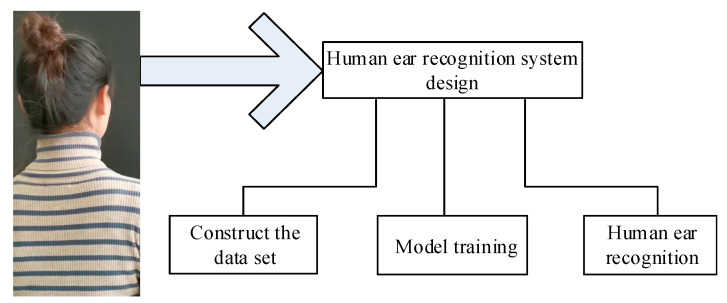
The block diagram of the dynamic human ear recognition system.

The block diagram of the dynamic human ear recognition system based on deep learning is shown in Figure 1. The system is divided into three parts: the dataset construction part, the model training part and the human ear recognition part.

The research process can be summarized as:

First, ear datasets including training sample sets and test sample sets were constructed.

Then, model training was used to train sample datasets to determine the weights of the deep learning models. In order to accelerate the training speed of the model, the transfer learning method was adopted.

Finally, the trained human ear recognition model was applied to perform ear recognition by using test sample sets.

## 3. Development of CCU-DE Small Sample Database

Due to the problem of insufficient dynamic human ear data, this paper establishes a small sample human ear database (CCU-DE). The data in the database consists of pictures and videos: Eardata1, Eardata2, Eardata3, Eardata4, and Eardata5. The basic idea of establishing this database was to simulate real scenes of real life as much as possible, taking into account that the human ear will perform multi-angle translational and rotational movements in front of the camera, and that human ears will be affected by changes in strong or weak light, such as sunlight, fluorescent lamps and curtains, and that the recognition of human ear is often interfered with by different life scenes such as earphones, glasses, hair, earrings, and so on.

### 3.1. Collection Tool

The camera device was an ordinary mobile phone camera. The parameters are shown in Table 1.

### 3.2. Acquisition Environment

The collection site of this database was an open space near the window of the laboratory, with a black wall in the background, which was divided into two types of illumination changes: strong light and low light. The strong light was an environment of sunlight and whole house light in the laboratory; the weak light was an environment of drawn curtains and half the house light in the laboratory.

In order to ensure that the lens of the mobile phone and the human ear were kept on a level surface, the mobile phone was placed on a tripod that could be adjusted up and down according to the height of the person being photographed, and the position of the tripod was fixed. The 0° vertical line was defined as a straight line between the wall and the lens, the distance between it and the lens was d = 1.2 m, and it was parallel to the lens. The shooting center was defined as a straight line passing through the center of the lens, perpendicular to the 0° vertical line, and the inter-section point with the 0° vertical line was the shooting center. In the movement area of the human body, we took the center of the shooting as the origin, marked the 0° and 90° rectangular coordinate axes with red tape, and marked the human movement route at other angles with green tape.

**Figure 2:**
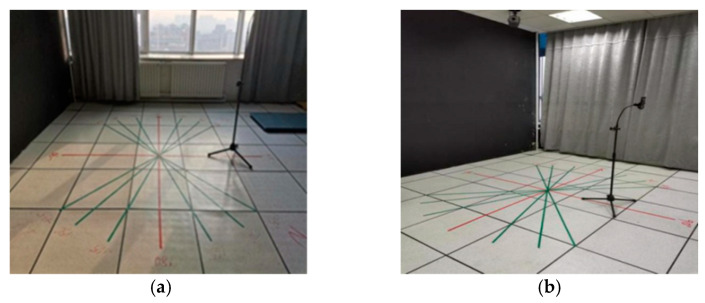
Collection environment of human ear database; (**a**) strong light; (**b**) weak light.

When shooting dynamic human ear videos, in order to make sure the dynamic human ears always appear in the shooting lens, the lens can be adjusted up and down according to the height of the person being photographed in the original position, and the left and right rotation movement can be carried out according to the range of the person being photographed in the lens. We ensured that the photograph centered in the lens, and each video was about 10 s in length. The collection environment is shown in Figure 2.

### 3.3. The Development of the Human Ear Database

After shooting, each person was numbered, and his/her name, gender, nationality, native place, date of birth age and other basic information were recorded in the form “CCU-DE Human Ear Database Information Collection Form”.

In CCU-DE small-sample database, there were 34 participants who were students at Changchun University, named from No. 1 to No. 34. They were aged between 21 and 29 (26% female and 74% male). 

In the CCU-DE database there were five databases: Eardata1, Eardata2, Eardata3, Eardata4 and Eardata5. The ear database information of each person is shown in Table 2. Each participant was photographed with 28 static images. No. 1, No. 27 to No. 32 were shot with 24 dynamic videos, while the others were shot with 22 dynamic videos, and the extra 2 videos were rotated 180°. Special attention was paid to the collection of 30 segments of dynamic ear videos of No. 33 (including 6 segments of dynamic ear occlusion videos with 0-degree linear back-and-forth translation).

#### 3.3.1. Eardata1

**Figure 3:**
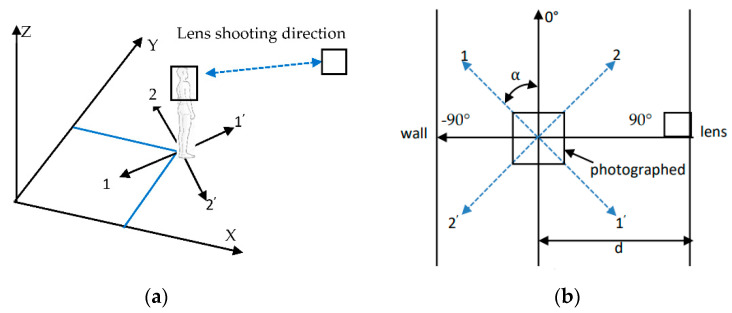
Static human ear fixed-point shooting; (**a**) 3D schematic diagram; (**b**) plane schematic diagram.

Under a simple black background, the subject stood on the shooting center, the human body was kept upright, the eyes were looking straight ahead, and the fixed-angle shoot was carried out. Figure 3a shows the 3D schematic diagram of the static human ear fixed-point shooting, and Figure 3b shows the plane schematic diagram.

In the process of rotating clockwise from the direction of position 2’ (−135°) to the direction of position 1’ (135°), we collected images from seven angles: the back left ear (−135°, −150°, −165°), right left ear (180°) and the front left ear (165°, 150°, 135°). We took one picture at each angle in strong light and low light. A total of 14 left ear images were collected for each subject. So, there were 34 people and 476 left ear pictures in Eardata1. Eardata1-left is shown in Table 3.

In the process of rotating clockwise from the direction of position 1 (−45°) to the direction of position 2 (45°), we collected images from seven angles: the back right ear (−45°, −30°, −10°), right ear (0°) and the front right ear (10°, 30°, 45°). We took one picture at each angle in bright light and low light. A total of 14 right ear images were collected for each subject. So, there were 34 people and 476 right ear pictures in Eardata1. Eardata1-right is shown in Table 4. So, Eardata1 had 952 images.

Eardata1 naming rule is personnel number + type (Static State, SS) + Strong Light (HL) or Low Light (LL) + Left Ear (LE) or Right Ear (RE) + rotation angle. For example, “1_SS_HL_RE_45” means that the right ear of the subject of No. 1 was rotated clockwise from position 1 to position 45° under strong light.

#### 3.3.2. Eardata2

**Figure 4:**
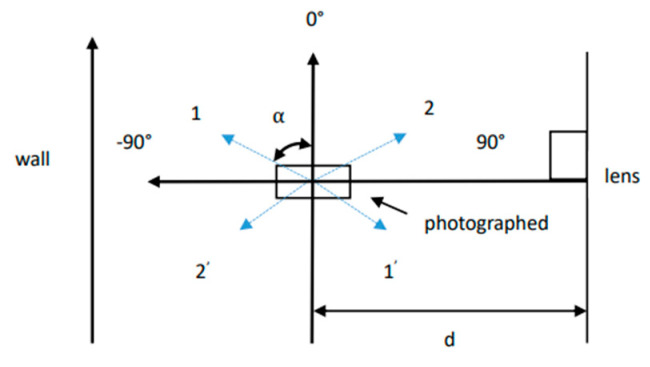
The translational motion of the human ear.

This database was a video segment for when the human ear moves with the human body when the subject is walking normally along different angles. The translational motion of the human ear simulates the translational motion trajectory of the human ear when the person moves in a straight line at different angles in front of the camera in real life. The schematic diagram of its trajectory is shown in Figure 4.

α is the angle between the 0° vertical line, and the direction of the photographed ear moves through a straight line. The person moves through the four straight lines of α = 135°, α = 150°, α = 165° and α = 180° from the shooting center. At this time, the camera records the translational motion video of the left ear at various angles. When the person moves through the shooting center along the four straight lines of α = 45°, α = 30°, α = 15°, and α = 0°, the camera records the translational motion video of the right ear at various angles. We collected eight videos of each photographed person’s two ears. So, there were a total of 16 videos, under both strong and weak illumination, as shown in Table 5. The number of people collected was 33. Therefore, Eardata2 contained 528 videos in 33 categories.

The Eardata2 naming rule was personnel number + type (Translational Motion, TM) + Strong Light (HL) or Low Light (LL) + Left Ear (LE) or Right Ear (RE) + translation angle. For example, “1_TM_HL_RE_45” means that the shooting information of the No. 1 photographed person proceeded dynamic translational movement under strong light conditions, starting from the bottom left along a straight line perpendicular to 0° at a 45° angle, and moved along the straight line through the shooting center to the top right.

#### 3.3.3. Eardata3

**Figure 5:**
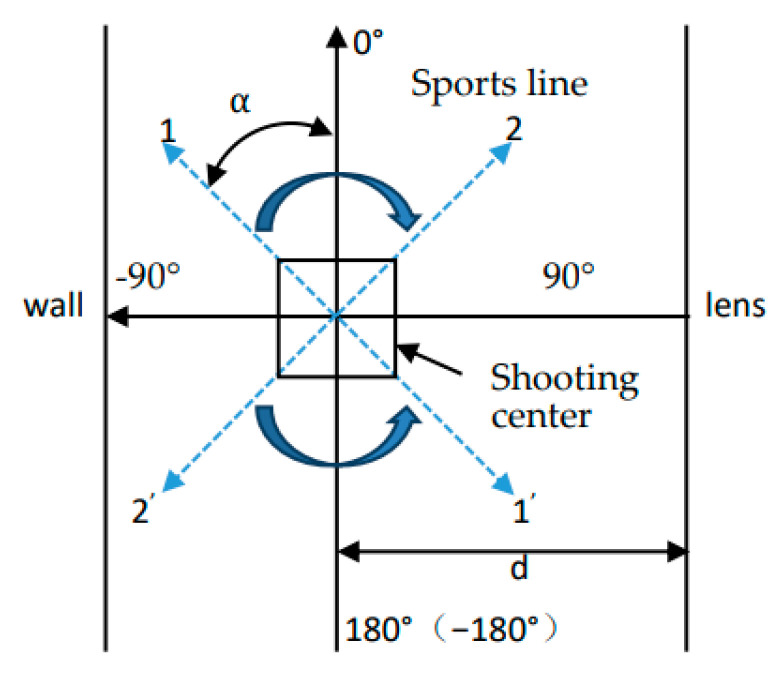
The rotation motion of the human ear.

Eardata3 refers to the video segment in which the subject stands on the shooting center and rotates at a constant speed of 90°. The human ear rotation motion simulates the human ear movement track when the subject turns around in front of the camera in real life, and its plane schematic diagram is shown in Figure 5.

The subject stood in the center of the shooting range and rotated at a uniform speed. The rotation angle was from −45° to 45°, that is, from P to Q for a video shot for the right ear. The rotation angle was from −135° to 135°, that is, from Q’ to P’ for a video shot for the left ear. Under each kind of light the human ear rotated and two videos were shot. Under the two kinds of light, there were four videos in total, as shown in Table 6.

The number of people collected in Eardata3 was 33, with numbers from 1 to 33. So, there were 132 videos.

The Eardata3 naming rule was personnel number + type (Rotation Motion, RM) + Strong Light (HL) or Low Light (LL) + Left Ear (LE) or Right Ear (RE). For example, “1_RM_HL_LE” refers to the video information of the subject No. 1 and whose left ear rotated 90° while standing in the shooting center and rotating counterclockwise from −135° to 135° under strong light.

#### 3.3.4. Eardata4

Eardata4 was the video segment for the subject standing on the shooting center and rotating at a constant speed of 180°. The video of the right ear movement was shot when the rotation angle rotated clockwise from −90° to 90°, and the video of the left ear movement was shot when the rotation angle rotated counterclockwise from −90° to 90°. The video was only shot under strong light. Two videos of each ear were collected. There were 8 participants with numbers from 27 to 33, and 1. Therefore, Eardata4 contained a total of 16 videos. The information of Eardata4 is shown in Table 7.

Eardata4 naming rule is personnel number + type (Rotation Motion, RM) + Strong Light (HL) + Left Ear (LE) or Right Ear (RE) + 180. For example, “1_RM_HL_LE_180” indicates that the video is the video information of the subject No. 1, whose left ear rotated 180° when the subject stood in the shooting center and rotated counterclockwise from −90° to 90° under strong light.

#### 3.3.5. Eardata5

Eardata5 is the dynamic human ear video segment with interference. Interference was used to simulate a situation in which ears were covered by earphones, glasses, hair, scarves, masks, ear studs, or other parts. Eardata5 mainly considers the interference of glasses, earphones, caps, ear studs and hair. In order to reduce the shooting time of the person being photographed, the interference conditions were determined according to the actual situation of the person being photographed. Interference information of Eardata5 is shown in Table 8.

Under strong light, the subjects were required to wear earphones, glasses or caps, and girls could cover part of the ears by disheveling their hair or wearing ear studs. The dynamic human ear occlusion videos of the subjects standing in the center of the shot were collected, showing the rotation of the left and right ears within 90° range and the straight back and forth movement of 0°. Among them, the rotation angle from −135° to 135° was the motion track for partial occlusion of the left ear, and the rotation angle from −45° to 45° was the motion track for partial occlusion of the right ear.

A total of 68 videos were collected from 34 people, and 2 videos were collected from each person. In addition, 6 videos of dynamic human ear occlusion with a 0° straight and back and forth translation motion of No. 33 human ears were captured, including 2 segments of glasses occlusion, 2 segments of “glasses + earphone” occlusion, and 2 segments of “glasses + earphone + cap” occlusion, as shown in Table 9.

Therefore, Eardata5 contained a total of 74 videos in 34 categories. The Eardata5 naming rule is personnel number + interference category (earphone, glasses, hair and cap) + movement type (Rotation Motion, RM or Translational Motion, TM) + Left Ear (LE) or Right Ear (RE). For example, “1_erephone_RM_RE” indicates that this video is the interference video information of the right ear rotating 90° during the shooting process of the subject No. 1 rotating clockwise from the −45° direction to the 45° direction under the interference condition of earphone.

## 4. Experimental Design

### 4.1. Datasets

From Figure 1 we can see that, in order to test the effectiveness of the deep learning model for dynamic human ear recognition, three datasets were used: (1) COCO [19], http://cocodataset.org; (2) USTB [14], http://www.ustb.edu.cn/resb; (3) CCU-DE. COCO and USTB datasets which are public and widely used in the field of deep learning and static ear recognition. CCU-DE is a static and dynamic ear database developed in this paper.

### 4.2. Experimental Setting

In order to test the practicability and effectiveness of the proposed CCU-DE small sample database, we set up two groups of experiments. The first group of experiments were based on the YOLOv3 deep learning model. The second group of experiments involved the comparison of the different deep learning models, such as YOLOv3, YOLOv4, YOLOv5, Faster R-CNN and SSD.

The experimental parameters were set as follows:

1. The computer was configured with 2.9 GHz Intel Core CPU and 16GB memory;
2. The software platforms were Python 3.8 and pytorch 1.8.

### 4.3. Evaluation Indicators

**Figure 6:**
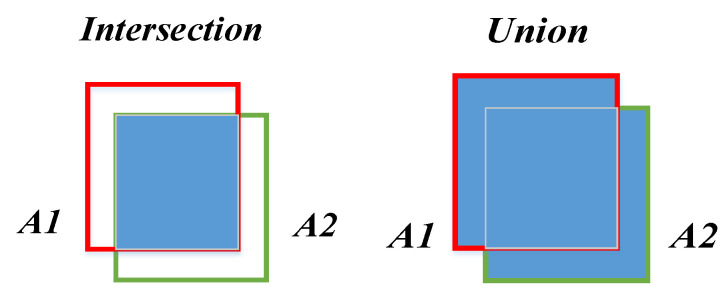
The schematic diagram of IOU.

The main evaluation indicators of the ear recognition model in this paper were:

1. Intersection and union (IOU):

The schematic diagram of IOU is shown in Figure 6. The green frame A2 represents the real detection frame, and the red frame A1 represents the prediction frame. The ratio of the intersection and union of the two is used to measure the degree of overlap between the prediction frame and the real frame. IOU is defined as Formula (1). When using IOU to judge whether the detection is correct, a threshold value needs to be set: the commonly used threshold value is 0.5.

IOU = (A1∩A2)/(A1∪A2)(1)

2. Classification target division result:

The test result will divide the classification target as true positives (TP), false positives (FP), false negatives (FN) and true negatives (TN).

3. Precision and Recall:

Precision = TP/(TP + FP)(2)

Recall = TP/(TP + FN)(3)

As shown by Formula (2), Precision is the proportion of samples that the classifier considers to be positive and is indeed positive to all the samples that the classifier considers to be positive. From (3), Recall is the proportion of all actual positive samples considered to be positive.

4. Average precision (AP):

Average precision is accuracy of a single-class model in Formula (2), mean average precision (mAP) is average recognition precision of all images in all categories.

### 4.4. Model Training

#### 4.4.1. Determination of the Initial Value of the Model

Using the dynamic human ear recognition model based on deep learning shown in Figure 1 for human ear recognition requires a large amount of human ear data to train the model. We adopted the method of transfer learning to determine the initial value of the model. Migration learning mainly uses the characteristics of a certain correlation between different classification tasks, which can not only reduce the training time of the model, but also improve the accuracy of deep learning.

We first used more than 330,000 images in 80 categories in the COCO dataset to determine the initial value of the model for deep learning models. Then, we used the 10 types 100 static ear pictures of database3 in the USTB to further determine the initial value of the model. Thus, the initial values of the dynamic ear recognition model of the CCU-DE small sample database were determined.

#### 4.4.2. The Effect of Epoch Value on the Training Model

In deep learning, the gradient descent method is often used for model training. An epoch refers to the time it takes to send all data into the network to complete a forward calculation and back propagation process. For training sets of different sizes and types, the required epoch size is also uncertain. In this paper, we conducted an experimental study on the influence of epoch value on the training model.

In the training of the deep learning model, the training dataset was a picture. The dataset in this paper was a dynamic video, so it was necessary to turn the video into a frame picture.

The training set in the experiment came from Eardata3 and Eardata4 in the CCU-DE small sample database. We randomly selected two types whose numbers were 25 and 26 in Eardata3. All eight categories in Eardata4 were selected. The purpose of the selection was to make the experiment more difficult, since Eardata4 was a 180° rotation and Eardata3 was a 90° rotation.

Ten types of dynamic human ear videos were sampled at two frames per second, and the first frame was taken to form the sample training set. The training samples are shown in Table 10: ‘ear1’ is the human ear category of participant No. 1, and ‘ear25’ to ‘ear33’ have the same definition. We defined NF as the number of frames for each type of human ear video, i.e., 153 NF in column 3 row 1 indicates that the left ear in the participant No. 1 video has 153 frames.

In the actual training process, a certain number of various samples in Table 10 were randomly selected to participate in the training. The number of samples participating in the training was 2063, and accounted for about 63% of the total number of samples: Batchsize = 12, learning_rate = 0.001. Different ear recognition models were trained by the initial value determined in Section 4.4.1, and were obtained by changing the epoch value. Then, 3274 pictures of all samples were used as the test set (including 2063 trained pictures and 1211 untrained pictures). The test results corresponding to different epoch values are shown in Table 11.

Comparing Table 10 and Table 11, we can see that the number of ground-truth (3956) in Table 11 is greater than the total number of samples in Table 10 (3274). This is because there were double ears in some human ear images in Table 10. It can be seen from Table 11 that when epoch = 1100, the TP value was the largest, and the FP value was relatively small. The AP of this model for human ear classification can reach 98.32%, and the average error rate was the lowest. Therefore, it can be seen that the epoch value had a greater impact on the training results of the model. In the model training module, it was necessary to test and compare the recognition results according to the number of sample categories and the total number of samples participating in the training to determine the appropriate epoch value to obtain an ideal training model.

### 4.5. Experimental Results Based on YOLOv3 Deep Learning Model and CCU-DE Datasets

#### 4.5.1. The Training Data Experiment Results

**Figure 7:**
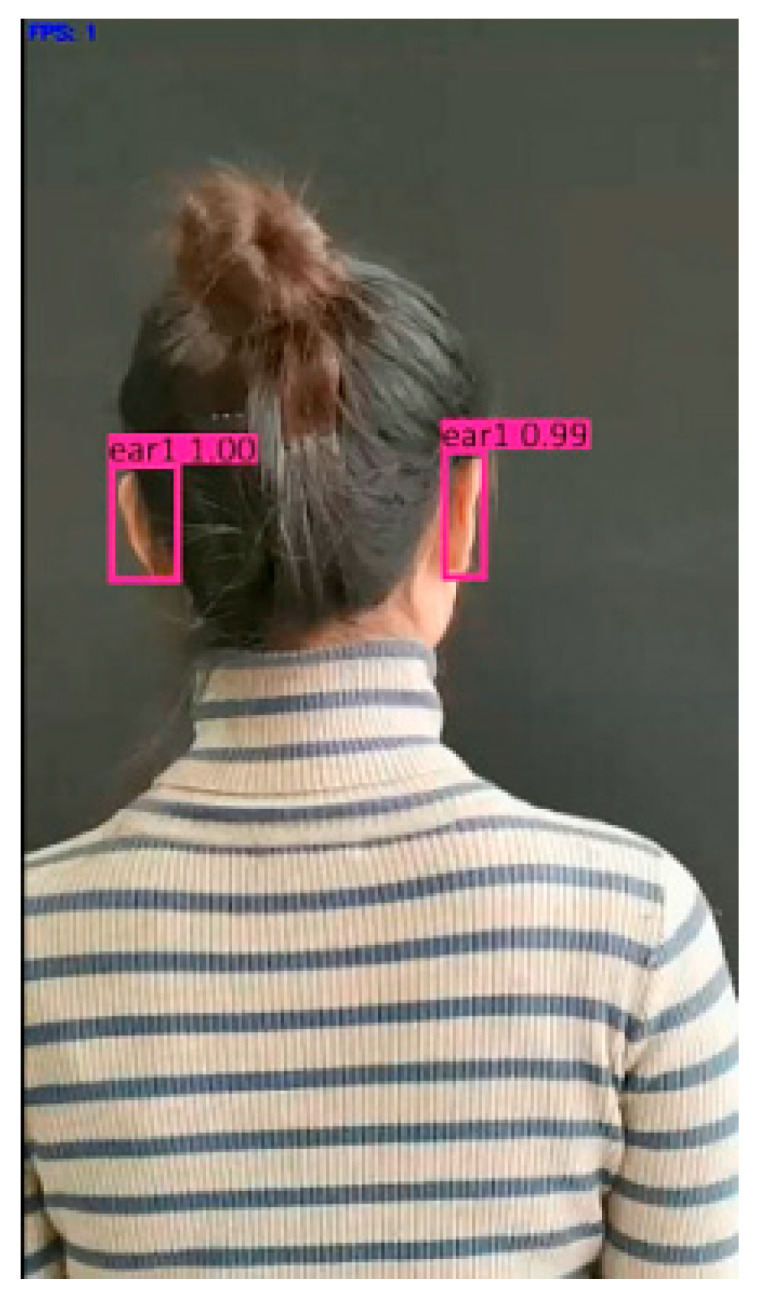
Video test results of ear1.

Using the ear recognition model at epoch = 1100 in Section 4.4.2, the 10 types of videos in Table 10 were tested. The screenshot result of ear1 test videos is shown in Figure 7. We can see that test videos could be correctly identified. The precision for the left and right ear of ear1 were 1.00 and 0.99, respectively.

**Figure 8:**
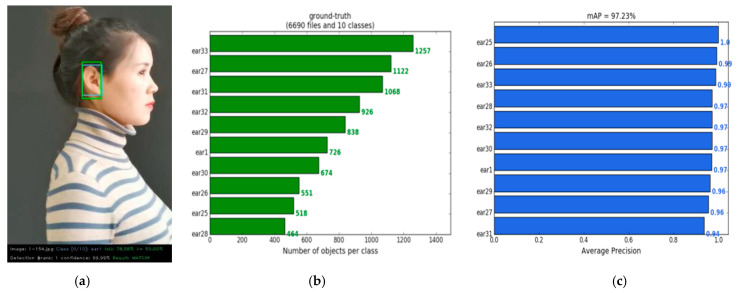
Performance index test results. (**a**) IOU; (**b**) the number of real frames of various human ear targets; (**c**) mAP.

In order to give a specific performance index evaluation, we took a screenshot of the video in this experiment as a test set and labelled it. The test video selection and frame cut are shown in Table 12. The test result is shown in Figure 8.

The IOU value of ear1 in Figure 8a was 78.58%, and greater than 50.00%, and therefore could be considered a real test. The final matching result was “MATCH”. That is, ear1 could be correctly identified. From Table 12 and Figure 8b we can see that the 6690 human ear test pictures in 10 categories in the test set contained a total of 8144 real frames. This is because some images contained both the left and right ears. Taking ear1 class as an example, the No. 1 participants’ video had 550 frames in Table 12, but the number of ear1 in Figure 8b was 726. The precision of ear1 was 0.97. Figure 8c gives average precision for each type, and mAP was 97.23%. From Figure 8c, we can also see that the precision of ear25 was 1.0 and the precision of ear26 was 0.99. So, we can conclude that the precision in Eardata3 rotated by 90° was greater than Eardata4 rotated by 180°.

#### 4.5.2. Different Contrast Control Experiments

Generally, the greater the contrast, the higher the image clarity, and the more vivid the color. Conversely, the lower the contrast, the worse the image clarity, and the detail in the image is worse. The contrast of all pictures in Table 12 underwent enhanced processing. The values of contrasts were 0.9, 0.8, 0.7 and 0.6. The results of different contrast control experiments and mAP experiments are shown in Table 13. From Table 13, we can see that, except for ear25 and ear26, the precision of all other ears decreased with decreasing contrast. The next step was to further increase the experimental volume to prove whether the reduction of contrast had an effect on the precision for all dynamic human ears. We can also conclude that contrast had a greater effect on Eardata4 than Eardata3.

#### 4.5.3. Contrast Experiment of Each Angle Posture of Translational Motion

The size of the translation angle has a certain impact on the human ear video recognition effect. Generally speaking, the smaller the translation angle, the better the recognition effect. Conversely, the larger the angle, the worse the recognition effect.

This experiment selected eight segments of dynamic human ear translation videos from different angles of ear1 in the untrained Eardata2 from the CCU-DE small sample human ear database under strong light conditions. The simulation results are shown in Table 14. It can be seen from the results that the smaller the translation angle, the higher the accuracy of human ear recognition. For untrained test sets, high recognition accuracy can also be achieved. So, the human ear recognition model in this paper is very robust to changes in the posture of each angle with translational motion.

#### 4.5.4. Rotational Motion Comparison Experiment

The experiment in this section was mainly used to simulate the situation in which the human body carries on rotational motion (RM) and drives the human ear to rotate. The experimental samples came from the untrained dynamic human ear video of ear1 under strong light conditions: 90° rotation in Eardata3, and 180° rotation in Eardata4. Each angle had one segment for the left and right ears, a total of four segments. The experimental results are shown in Table 15. It can be seen from the experimental results that the rotation movement had a certain impact on the recognition results of human ears. The greater the rotation angle, the slightly lower the mAP value. However, the mAP value of 180° rotation was above 98%, which could meet actual needs. This further illustrates that the ear recognition model proposed in this paper has certain robustness.

#### 4.5.5. Comparison Experiment with and without Occlusion

**Figure 9:**
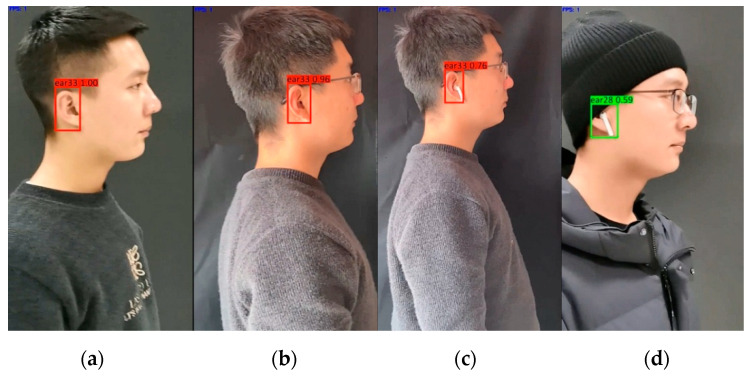
Experimental results of mAP with different occlusion: (**a**) unobstructed; (**b**) glasses; (**c**) glasses and earphones; (**d**) glasses, earphones and cap.

In practical applications, human ears are often occluded in front of the lens, so the robustness of the human ear recognition model is required to be high. Common occlusions include glasses, earphone, cap and hair, etc. In this section of the experiment, common features of video were a 0° round-trip translation with untrained person No. 33. We selected two videos of Eardata2 under unobstructed conditions and six videos of Eardata5 with glasses, glasses and earphones, and glasses, earphones and a cap. The experimental results are shown in Figure 9. The precision of No. 33 without occlusion was 1.00, as seen from Figure 9a. The precision of No. 33 with glasses was 0.96 (Figure 9b). The precision of No. 33 with glasses and earphones was 0.76 (Figure 9c). In Figure 9d, ear33 was recognized as ear28. So, it can be seen from the experimental results that the larger the area of human ears occluded, the lower the recognition rate.

### 4.6. Comparative Experiment of CCU-DE Datasets and Different Deep Learning Models

**Figure 10:**
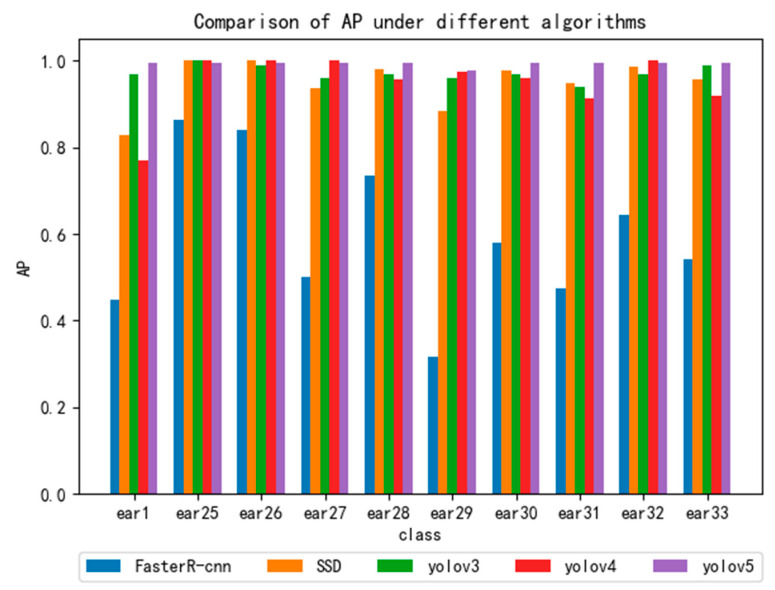
Comparative experiment of YOLOv3, YOLOv4, YOLOv5, SSD and Faster R-CNN models.

In order to further verify that different deep learning models can also be used for dynamic human ear recognition by using the CCU-DE database, we conducted comparative experiments on the different deep learning models. In the experiment, mainstream YOLO series models such as YOLOv3, YOLOv4 and YOLOv5, Faster-R-CNN, and SSD deep learning models were adopted. Figure 10 displays the experimental results of the five models. The results show that the CCU-DE small sample database could be tested by using the different deep learning models and had a high test accuracy.

## 5. Conclusions

In this paper, we came to three conclusions. (1) We developed a small-sample human ear database of CCU-DE, containing five human ear databases. CCU-DE fully considers the various complex situations and posture changes of human ear images, such as translation angle, rotation angle, illumination changes, occlusion and interference (glasses, earphones, caps, etc.) and other factors, making the research of dynamic ear recognition more realistic, and increasing the applicability of dynamic ear recognition. (2) The block diagram of the dynamic ear recognition based on deep learning was designed. We adopted the method of transfer learning to determine the initial value of the model, because this can not only reduce the training time of a model, but also improve the accuracy of deep learning. (3) We used different deep learning models to test the CCU-DE small sample database. The experiments we did are shown in Table 16. The experimental results showed that CCU-DE can be used in studies of dynamic human ear recognition, and it is more conducive to the application of dynamic human ear recognition in real life.

## Figures and Tables

**Table 1:** Related parameters of database.

Parameters	Value
Camera	HONOR20
Frame rate	30
Video resolution	[16:9] 720p 1280*720
Video format	MP4
Image Resolution	3000*4000
Image format	JPG
Focal length	2.0

**Table 2:** Human ear database information.

Database	Shooting Situation
Eardata1	Static human ear fixed-point shooting
Eardata2	Video of human ears moving in translation with the human body when the human body is walking normally at different angles
Eardata3	Video of the photographed standing on the shooting center doing a 90° uniform rotation motion
Eardata4	Video of the photographed standing on the shooting center doing a 180° uniform rotation motion
Eardata5	Dynamic human ear video with the interference information

**Table 3:** Human ear database information of Eardata1-left.

	Eardata1-Left													
Illumination	Strong Light							Low Light						
Angle (°)	−135	−150	−165	180	165	150	135	−135	−150	−165	180	165	150	135
Number of pictures	1	1	1	1	1	1	1	1	1	1	1	1	1	1
Total	14 × 34													

**Table 4:** Human ear database information of Eardata1-right.

	Eardata1-Right													
Illumination	Strong Light							Low Light						
Angle (°)	−45	−30	−10	0	10	30	45	−45	−30	−10	0	10	30	45
Number of pictures	1	1	1	1	1	1	1	1	1	1	1	1	1	1
Total	14 × 34													

**Table 5:** Human ear database information of Eardata2.

	Left Ear								Right Ear							
Illumination	Strong Light				Low Light				Strong Light				Low Light			
Angle (°)	135	150	165	180	135	150	165	180	45	30	15	0	45	30	15	0
Number of videos	1	1	1	1	1	1	1	1	1	1	1	1	1	1	1	1
Total	16 × 33															

**Table 6:** Human ear database information of Eardata3.

	Left Ear		Right Ear	
Illumination	Strong Light	Low Light	Strong Light	Low Light
Angle (°)	−135∼135	−135∼135	−45∼45	−45∼45
Number of videos	1	1	1	1
total	4 × 33			

**Table 7:** Human ear database information of Eardata4.

	Left Ear	Right Ear
Illumination	Strong Light	Strong Light
Angle (°)	−90∼90 (counterclockwise)	−90∼90 (clockwise)
Number of videos	1	1
Total	2 × 8	

**Table 8:** Interference information of Eardata5.

Total Number of People	Type	Interference Category	Participants
34	Single	Earphone	No. 4, 6, 9, 10~14, 17, 20~22, 25, 26, 28, 30
		Glasses	No. 5, 7, 8, 15, 16, 18, 19, 23, 24, 27, 29, 31
		Hair	No. 1, 32
		Cap	No. 34
	Overlap	Earphone + glasses	No. 2
		Glasses + hair + ear stud	No. 3
		Glasses + earphone + cap	No. 33
1	Single	Glasses	No. 33
	Overlap	Glasses + earphone + cap	No. 33
		Glasses + earphone	No. 33

**Table 9:** Human ear database information of Eardata5.

	Left Ear		Right Ear	
Illumination	Strong Light		Strong Light	
Angle (°)	−135∼135	0	−45∼45	0
Number of videos	1	3	1	3
Total	2 × 34 + 6			

**Table 10:** Training sample set.

Database	Category	Left Ear (NF)	Right Ear (NF)	Total (NF)
Eardata4	ear1	153	135	288
Eardata3	ear25	111	139	250
Eardata3	ear26	112	154	266
Eardata4	ear27	249	156	405
Eardata4	ear28	96	106	202
Eardata4	ear29	163	188	351
Eardata4	ear30	156	124	280
Eardata4	ear31	203	186	389
Eardata4	ear32	175	182	357
Eardata4	ear33	153	251	486
Total		1653	1621	3274

**Table 11:** The results of various indicators in different epochs.

Epoch	Ground-Truth (NF)	TP (NF)	FP (NF)	AP (%)	Log-Average Miss Rate
800	3956	3844	73	96.99	0.031
900	3956	3848	28	97.17	0.029
1000	3956	3521	193	88.52	0.132
1100	3956	3890	57	98.32	0.018
1200	3956	3859	73	97.46	0.026
1300	3956	3759	278	94.82	0.054

**Table 12:** The test video selection and frame cut.

Database	Category	Left Ear (NF)	Right Ear (NF)	Total (NF)
Eardata4	ear1	298	252	550
Eardata3	ear25	231	287	518
Eardata3	ear26	234	317	551
Eardata4	ear27	508	321	829
Eardata4	ear28	201	222	423
Eardata4	ear29	336	385	721
Eardata4	ear30	321	257	578
Eardata4	ear31	415	382	797
Eardata4	ear32	360	373	733
Eardata4	ear33	479	511	990
Total		3383	3307	6690

**Table 13:** Experimental results of mAP with different contrasts.

Contrast	ear1	ear25	ear26	ear27	ear28	ear29	ear30	ear31	ear32	ear33	mAP (%)
1	0.97	1.0	0.99	0.96	0.97	0.96	0.97	0.94	0.97	0.99	97.23
0.9	0.95	1.0	0.99	0.95	0.95	0.95	0.97	0.95	0.97	0.99	96.89
0.8	0.94	1.0	0.99	0.95	0.95	0.95	0.97	0.94	0.97	0.98	96.37
0.7	0.93	1.0	0.99	0.95	0.93	0.94	0.97	0.91	0.96	0.98	95.47
0.6	0.90	1.0	0.99	0.94	0.82	0.91	0.95	0.90	0.90	0.97	92.89

**Table 14:** The experimental results of different angle TM.

Angle	Ground-Truth	TP (NF)	FP (NF)	mAP (%)	Log-Average Miss Rate
135	225	190	64	84.44	0.16
150	276	269	7	97.46	0.03
165	248	247	18	99.60	0.00
180	232	232	0	100.00	0.00
0	241	241	0	100.00	0.00
15	273	273	0	100.00	0.00
30	286	281	0	98.25	0.02
45	282	274	3	97.16	0.03

**Table 15:** The experimental results of different angle RM.

Angle	Ground-Truth	TP (NF)	FP (NF)	mAP (%)	Log-Average Miss Rate
90	412	411	6	99.72	0.00
180	718	706	12	98.23	0.02

**Table 16:** The experiment in this paper.

The Experimental Project	Database	Whether the Test Set Was Trained	Deep Learning Model	Section
Influence of training parameters	Eardata3, Eardata4	Trained	YOLOv3	Section 4.4.2
The training data experiment	Eardata3, Eardata4	Trained	YOLOv3	Section 4.5.1
Influence of contrast	Eardata3, Eardata4	Untrained	YOLOv3	Section 4.5.2
Influence of translational motion angle	Eardata2	Untrained	YOLOv3	Section 4.5.3
Influence of rotation motion angle	Eardata3, Eardata4	Untrained	YOLOv3	Section 4.5.4
Experiment with and without occlusion	Eardata2, Eardata5	Untrained	YOLOv3	Section 4.5.5
Comparison of different deep learning models to test CCU-DE	Eardata3, Eardata4	Untrained	YOLOv4, YOLOv5, Faster R-CNN, SSD	Section 4.6

## Data Availability

(1) COCO: http://cocodataset.org. (2) USTB: http://www.ustb.edu.cn/resb. (3) CCU-DE has just been established and is still being perfected. The videos in the database contain a large amount of biometric information other than the human ear. Although written authorization from the participants has been obtained, no links to the archived datasets are publicly available at present. If you need to do related research on human ear recognition, you can contact the author of this article to use the database. Email: 0451lym@163.com and qjr1107007169@163.com.

## Abbreviations

**Abbreviations**	**Acronyms**
CCU-DE	the Changchun University-Dynamic human Ear database
YOLO	You Only Look Once
SSD	Single Shot MultiBox Detector
CNN	Convolutional Neural Network
Faster R-CNN	Faster Regions with CNN
COVID	Corona Virus Disease
SS	Static State
HL	Strong Light
LL	Low Light
LE	Left Ear
RE	Right Ear
TM	Translational Motion
RM	Rotation Motion
TP	true positives
FP	false positives
FN	false negatives
TN	true negatives
AP	average precision
mAP	mean average precision
